# What do patients think about the Hungarian health care system and medical students’ learning outcomes? A cross-sectional study on the patients’ perspective in Hungary

**DOI:** 10.1186/s12909-023-04350-w

**Published:** 2023-05-25

**Authors:** Zsuzsanna Varga, Zsuzsanna Pótó, Árpád Csathó, Zsuzsanna Füzesi

**Affiliations:** Department of Behavioral Sciences, Medical School, University of Pécs, Szigeti str. 12. 7624, Pécs, Hungary

**Keywords:** Medical teacher, Pedagogical skills, Gap matrix, Patients’ expectation, Patients’ satisfaction

## Abstract

**Background:**

The doctor-patient relationship has changed a lot in the 21st century and the varying expectations of the patients play an important role in future professional medical care. The knowledge of patients’ needs is crucial in determining the learning outcomes in medical education. The objective of this study was to examine the expectations of the patients regarding professional and soft skills (e.g. communicational skills, empathy) of doctors and to get a deeper view.

**Methods:**

Face-to face data collection through self-reported questionnaire in accredited health care institutions (GPs, hospitals, outpatient care) in Hungary was carried out in 2019. Descriptive statistics, independent sample t-tests, k-means cluster and gap matrices were performed to analyze the data.

**Results:**

In total 1115 patients (male-female: 50–50%, age groups: between 18 and 30: 20%, between 31 and 60: 40% above 60: 40%) participated in the survey. They rated sixteen learning outcomes along with two dimensions: importance and satisfaction. Except for one learning outcome, patients rated the outcomes more important than they were satisfied with them (negative gap). Positive gap was registered only in the case of respecting individual specialty during patient care.

**Conclusions:**

The results suggest the importance of learning outcomes in relation to the rate of satisfaction from the patients’ perspectives. In addition, the results support that patients’ need are not met in medical care. Patients’ ratings also make an emphasis on the fact that besides professional knowledge other learning outcomes are also important in health care which should have been emphasized as a basis in medical education.

## Background

Medical education has a key role in the later practice, attitude, and behavior of a medical doctor due to the global changes in medical knowledge and the varying expectations of the patients. Its goal is to educate physicians who are experts in the profession as well as take individual and collective responsibility towards society [1]. How can this goal be granted?

The understanding of quality from the perspective of consumers has been more and more crucial in formulating health care services and processes [1]. Laing emphasizes a need for reform of healthcare systems in western economies about the emphasis on reorienting service around the patient. In line with this, healthcare organizations shifted their goals to re-appraise the design of the service to take account of the changing patient expectations [2]. Patients have both individual and collective engagement in forming health care e.g. by giving feedback about health care services. The movement toward actively managing consumer perceptions of health care quality is important. To gain insight into the expectations of patients the Hastings Center made a report in 1998 summarizing their views on society beliefs and revealing that the cure of disease was the major expectation, and all other aspects of medicine were subordinated to this purpose [3]. Nowadays a need for a broader view can be recognized and physicians must be compassionate and empathetic in caring for patients [3–5]. According to the Association of American Medical Colleges Report establishing learning outcomes to guide the design, content and conduct of an educational program is an important principle in medical education [3]. These learning outcomes can ensure the above-mentioned goal and the quality of medication education. “The Dutch Blueprint” [4] created in 1994 was regarded as a pioneer in Europe, which summarizes the medical learning outcomes in a comprehensible system. In 2009, the outcomes and standards for undergraduate medical education by General Medical Council suggests that „good doctors make the care of their patients their first concern: they are competent, keep their knowledge and skills up to date, establish and maintain good relationships with patients and colleagues, are honest and trustworthy, and act with integrity” [5]. In the United States of America 1998 was the date of publishing the document listing 30 learning outcomes [3] in medical education. In Hungary a law determines the learning outcomes valid for medical students which was ratified in 2016. This summarizes the key knowledge, skills and attitudes medical students are expected to acquire by the time they qualify as doctors [6].

A lot of research has dealt with the goal of medical education. Most of them suggest [3, 4, 7, 8] that the goal of medical education is to train physicians who are prepared to serve fundamental purposes of medicine and the central promise is to improve the quality of care. On the one hand, if medical education is to serve the goal of medicine, then medical teachers must develop learning objectives, learning outcomes for medical education programs that improve these attributes [4]. Varga et al. [6] analyzed the attitudes of medical teachers (professors and medical doctors in a teaching position) towards learning outcomes in Hungarian medical schools. The results suggested that teachers ranked the learning outcomes (e.g. the professional practice needed for the everyday work, handling patients as equals and with respect, respecting human dignity of the patients and the relatives during patient care) with high importance and they considered it to be useful in the everyday job of a doctor. The results also imply the importance of the development of teachers’ skills.

The quality of medical education and its learning outcomes are closely linked to the success of patient care and patient satisfaction. The investigation of patients’ view about the learning outcomes may therefore provide highly valuable information for future developments of the medical curriculum. To our knowledge, however, such a study has not yet been carried out. In the present study, we therefore aimed to examine this question, and compare the results with those of similar studies examined the learning outcomes from teacher and student perspectives. Our main questions were that from a patient perspective, which learning outcomes are possibly more important, and which are less so, and how satisfied the patients are with the aspects of healthcare addressed by the specific outcomes.

More specifically, the objective of this study was in general to examine the expectations of the patients regarding professional and soft skills (such as communicational skills, empathy) of doctors and thus to get a deeper view understanding of this problem. In our survey we asked patients whether they were satisfied with the everyday job of a doctor in terms of the learning outcomes prescribed by the Hungarian law. Our intent was to raise an awareness of the importance of learning outcomes in medical education and give a view on the expectations of the patients in health care. Similar to Varga et al. previous analyses dealing with medical teachers’ importance and satisfaction rates regarding learning outcomes [6], the aim was to investigate patients’ importance and satisfaction rates of the learning outcomes during health care processes.

## Methods

### Study design, questionnaire

In Hungary there has not been developed a questionnaire specifically addressed to the attitudes of patients regarding the learning outcomes. Therefore, we used a modified version of the questionnaire developed by Varga et al. [6].

We adapted the twenty-five-item of Varga et al’s two questionnaires, one developed for the medical teachers and one for the medical students with the aim of preparing a new questionnaire specifically for patients regarding their attitudes towards the learning outcomes [6] containing soft skills, practical and theoretical knowledge, and respect towards patients. We constituted a committee of 16 experts (2 educational professionals, 2 sociologists, 3 clinicians, 1 linguist and 8 representatives of patients) who took part in the adaptation of the questionnaires to patients. The committee examined the twenty-five learning outcomes used in the questionnaire from the patients’ point of view. The committee selected eighteen learning outcomes as potentially relevant, so can be rated by patients based on their experiences spent in the health care system. The Content Validity Ratio (CVR) [9] was calculated by using the experts’ comment and scoring the questionnaire outcomes. Sixteen outcomes had the CVR bigger than 0.49 (e.g. agreement that the outcome was essential reached 80%), and so were accepted as items in final version of the questionnaire listed in Table 1 such as ‘the flexible professional and everyday thinking’, ‘treating the emotional reactions of the patients and relatives during patient care’ and ‘fully informing patients about their diseases’. The following two items with a maximum of 0.49 CVR were not included: ‘good time management’, ‘participation in further educational courses’. In the final questionnaire each of the outcomes were rated along with two dimensions: importance and satisfaction. The importance dimension reflects a view on the relevance of the learning outcomes in health care process. The satisfaction dimension reflects the experience of patients. These dimensions made it also possible to interpret the patients’ ratings in gap matrices. Patients rated the learning outcomes on a Likert-scale regarding whether they were important in the everyday work of a doctor (rating scale: 1 = the least important, 5 = the most important) and to what extent patients were satisfied with them during health care (rating scale: 1 = not at all, 5 = greatly). Specifically, patients were asked to answer for two questions as follows.

**Table 1:** Sample characteristics

Variables	Characteristics	nr. (%)
**Place of interview**	General practitioner	461 (41.3%)
	Outpatient care	291 (26.1%)
	Hospitals, clinics – Departments of Surgery and Internal Medicine	363 (32.6%)
**Gender**	Male	549 (49.2%)
	Female	566 (50.8%)
**Age**	18–30 years	216 (19.4%)
	31–60 years	448 (40.2%)
	61–93 years	451 (40.4%)
**Place of residence**	Village	50 (4.5%)
	Town	309 (27.7%)
	City	756 (67.8%)
**Level of education**	Primary school	109 (9.8%)
	Secondary school	715 (64.1%)
	College/university	267 (23.9%)
	Other	24 (2.2%)
**Frequency of doctor visits**	Maximum two times a year	284 (25.4%)
	Several times a year	343 (30.8%)
	Once a month	353 (31.7%)
	Once a week	75 (6.7%)
	Several times a week	60 (5.4%)

The table shows the patients’ characteristics (place of interview, gender, age, place of residence, level of education, frequency of doctor visits)

**Table 2:** Comparison (independent t-test) of importance and satisfaction of physicians’ learning outcomes by the patients

Learning outcomes	Importance N = 1115		Satisfaction N = 1115		Difference/gap (satisfaction-importance)	t
	Mean	SD	Mean	SD		
The timely theoretical and practical knowledge to the everyday work	4.86	0.43	4.34	0.77	-0.52	21.43**
The professional practice needed for the everyday work	4.82	0.50	4.32	0.80	-0.50	18.95**
The flexible professional and everyday thinking	4.70	0.56	4.01	0.95	-0.69	22.85**
Respecting human dignity of the patients and the relatives during patient care	4.79	0.50	3.94	1.03	-0.85	26.07**
Respecting the different demographic (sex, age), social and economic characteristics during patient care	4.27	0.96	3.97	1.00	-0.30	7.92**
Respecting individual specialty during patient care (e.g. familiar background, emotional state, sexual orientation)	3.31	1.54	3.71	1.17	0.40	-7.88**
Treating the emotional reactions of the patients and the relatives during patient care	4.18	0.96	3.79	1.10	-0.39	9.81**
Giving information suitable to the patients’ qualification, cultural background, cognitive state	4.83	0.51	4.00	1.05	-0.83	24.10**
Fully informing patients about their diseases	4.80	0.52	4.00	1.05	-0.80	23.58**
Establishing long term “partnerships” with patients (mostly with chronical diseases)	4.61	0.64	4.14	0.92	-0.47	15.89**
Handling patients as equals and with respect	4.85	0.43	4.10	1.00	-0.75	23.00**
An ongoing positive and motivated approach to work	4.66	0.61	3.93	1.05	-0.73	22.71**
The individual problem-solving skills (creativity) during everyday work	4.47	0.77	3.93	1.02	-0.54	15.71**
Handling appropriately patients’ expectations on therapy	4.43	0.78	3.96	0.99	-0.47	13.90**
Handling conflicts within the educational team and with the patients (and relatives)	4.34	0.83	3.94	0.99	-0.40	11.88**
Improving emotional intelligence	4.32	0.88	3.89	1.04	-0.43	11.61**

Note. ** p < 0.01

The table shows the importance and satisfaction of the learning outcomes for the patients’ perspective (mean importance, satisfaction and the standard deviations). The analysis shows that the importance of learning outcomes is rated all higher (except in one learning outcome: respecting individual specialty during patient care (e.g. familiar background, emotional state, sexual orientation) than their rate of satisfaction

1. How important do you consider the listed learning outcomes in the everyday job as a doctor?
2. To what extent are you satisfied with the listed learning outcomes during health care?

We also tested the face validity of the items. Clarity of wording of the questions stayed in focus in this phase. Thirty patients who did not participate later in the study were asked to evaluate our questionnaire items. Comments were received regarding on linguistic and semantic aspects, such as usage of words, synonyms. Patients recommended us to use more examples in the questionnaire to make it more comprehensible. Therefore, we clarified the questions with more examples as e.g. sex, age, and weight were used to indicate the different demographic, social and economic characteristics during patient care. Cronbach’s alphas indicated good level of reliability (importance: 0.86, satisfaction: 0.96; N = 30).

For the final, surveyed data (N = 1115) the Cronbach’s alphas were 0.81 (importance) and 0.96 (satisfaction) indicating a good-to-excellent level of reliability. According to the KMO values (KMO_I_=0.885; KMO_S_=0.960) and Bartlett-test significances (p < 0.001) the selected item groups of the importance of the learning outcomes and the satisfaction with the learning outcomes were suitable for factor analysis. So, the percentage of the total variance explained are appropriate (TVE_I_=52.3%; TVE_S_=67.2%). Based on results of the face, linguistic validity, the reliability, internal consistency (Cronbach’s alphas) and the factor analysis we considered the questionnaire a reliable instrument for this study.

Face-to-face data collection through self-reported questionnaire in accredited health care institutions (general practitioners, hospitals, outpatient care) in Hungary was carried out from July 8 to August 6, 2019. The interviews lasted for approximately 30 min in Hungarian language. In a cross-sectional design, 1213 patients were interviewed by fourteen students (as interviewers), who had active legal status in the University of Pécs, Medical School. Before the interviews the students got a training led by the Committee to ensure the same interview parameters during the survey.

Ethical approval for this study was granted by the Regional Ethical Committee of the University of Pécs (7632-PTE 2019).

### Participants

The participants were recruited in accredited health care places in Hungary on weekdays between 9 and 11 am. The respondents were explained the aim and procedures of the survey and were reassured about the anonymity of the questionnaire and the subsequent aggregate data analysis. All patients were asked to take part in the survey based on convenience sampling. Interviews with the patients were conducted face-to-face on a voluntary basis, anonymously. Written informed consents were obtained from the participants. Reasons for exclusion were severe health status of the patients (e.g. severe acute pain) and exceeding the determined quotas (male-female: 50–50%, age groups: between 18 and 30: 20%, between 31 and 60: 40% above 60: 40%).

### Statistical analysis

We used independent sample t-test (two-sided), gap matrices to analyze the data. The gap matrix was used to demonstrate the difference between the importance of the learning outcomes and the rate of satisfaction according to the patients. Generally, if the matrix shows a negative gap, it means that the learning outcomes are under the diagonal, so in our case patients consider them more important than they are satisfied with them. As opposed to the negative gap, a positive gap means that the patients are more satisfied with the learning outcomes than they rated their importance. Optimal performance is signaled by zero gap in gap matrices. In this case there is a balance between the rate of importance and satisfaction.

We also carried out an additional analysis. More specifically, a K-means cluster analysis was performed to get a profile of the patient characteristics in terms of the importance of the learning outcomes, satisfaction with them, and background/demographic variables.

## Results

In total 1115 patients participated in the survey, because 98 patients refused to take part in the survey referring to short of time, being in a hurry or being reluctant to answer the questions. According to the sample characteristics depicted in Table 2 most of the interviews were taken at general practitioners, the proportion of genders was balanced, the majority of the patients were over 30 years, lived in cities and had secondary school level of education and visited the doctors 3–12 times a year.

Interviews between the interviewer and the patients took place at bedside (in the hospitals and clinics) and in a separate room (in the GPs and at outpatient care) without being disturbed. They rated sixteen learning outcomes along with two dimensions: importance and satisfaction.

**Fig. 1:**
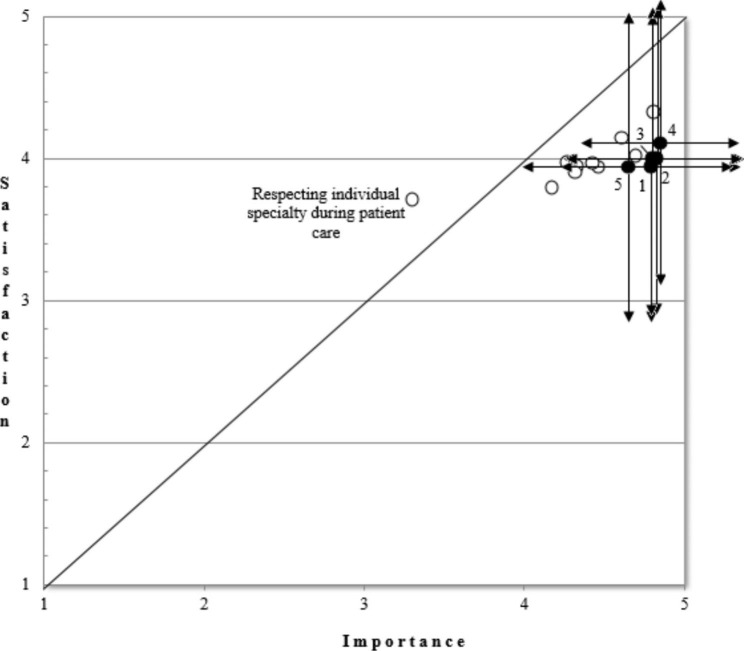
Gap of the perceived importance of and satisfaction with the learning outcomes according to the patients The matrix shows that fifteen learning outcomes are rated more important than its rate of satisfaction the patients (mean; importance, satisfaction). The arrows show the standard deviation of the ratings of importance and of satisfaction for the given learning outcome The mean ratings and the standard deviation of the following items are shown in parentheses. Maximum of importance and satisfaction: The timely theoretical and practical knowledge to the everyday work (4.86; 4.34, difference: -0.52). Minimum of importance and satisfaction: Respecting individual specialty during patient care (e.g. familiar background, emotional state, sexual orientation): (3.31; 3.71, difference: 0,40) The only positive GAP: Respecting individual specialty during patient care (e.g. familiar background, emotional state, sexual orientation): (3.31; 3.71, difference: 0,40) We registered the biggest gaps in the following learning outcomes: Respecting human dignity of the patients and the relatives during patient care (-0.85) Giving information suitable to the patients’ qualification, cultural background, cognitive state (-0.83) Fully informing patients about their diseases (-0.80) Handling patients as equals and with respect (-0.75) An ongoing positive and motivated approach to work (-0.73)

Table 1 indicates descriptive statistics, mean differences (i.e. gaps) between satisfaction and importance ratings as well as the results of independent t-test for the comparison of satisfaction and importance for each learning outcome. Figure 1 demonstrates the results of the gap analysis.

The mean ratings were high both in terms of importance and satisfaction: the mean values were ranged between 3.31 (‘respecting individual specialty during patient care (e.g. familiar background, emotional state, sexual orientation)’) and 4.86 (‘the timely theoretical and practical knowledge to the everyday work’) for importance, and between 3.71 (‘respecting individual specialty during patient care (e.g. familiar background, emotional state, sexual orientation)’) and 4.34 (‘the timely theoretical and practical knowledge to the everyday work’) for satisfaction. More specifically, out of the sixteen learning outcomes tested in this study seven had a mean importance rating over 4.0, the others were also above 3.0 indicating that participants considered each of the learning outcomes highly important. Based on the descriptive statistics the most important learning outcome was the ‘timely theoretical and practical knowledge to the everyday work’. Similar to importance, patients’ satisfaction with the implementation of learning outcomes during health care reached a high level as indicated by the high satisfaction scores having a mean above 3.5 for each. Patients were most satisfied with the same outcome, which they also considered the most important: ‘timely theoretical and practical knowledge to the everyday work’. Besides ‘the timely theoretical and practical knowledge to the everyday work of doctors’ ‘the professional practice needed for the everyday work’ got the highest satisfaction scores. ‘Establishing long term “partnerships” with patients’ reached also high satisfaction level suggesting that patients have satisfying partnership with their doctors, as well as they are satisfied with their theoretical and practical knowledge.

Although patients rated both satisfaction and importance with high scores, t-test revealed significant difference between importance and satisfaction for each outcome.

As shown in Table 1; Fig. 1, only one significant positive gap (the outcome of ‘individual specialty during patient care: e.g. familiar background, emotional state, sexual orientation)’ was observed (being above the diagonal on the matrix in Fig. 1, and a positive mean difference in Table 1). This positive gap suggested that patients’ satisfaction with the doctor’s ability to take account of individual differences during patient care was higher than patients felt this learning outcome important.

In fifteen cases, however, negative gap was observed suggesting that these learning outcomes were ranked by the patients more important than they were satisfied with them during health care. All these negative gaps were significant as indicated by the t-test.

A non-hierarchical cluster analysis was carried out using standardized scores (Table 3), k-means cluster, to create groups of patients with similar learning outcomes and sociodemographic background in the sample. We performed the analysis with two to five clusters and selected the cluster result based on the following criteria: the clusters significantly differ in all the 37 analyzed variables (using a one-way ANOVA), the clusters have at least 100 number of participants. We selected the four-cluster result of the analysis, because that had the best fit with the criteria (see Table). One-way ANOVA revealed a significant effect of clustering on each variable. The range of F value = 9.108–33.113, p < 0.001.

**Table 3:** Descriptive results of the k-means cluster analysis (mean, SD, %)

Variables	Clusters			
	1	2	3	4
N	317	252	377	145
Grand mean of importance over the 16 learning outcomes (SD)	4.37 (0.24)	4.60 (0.26)	4.83 (0.17)	3.86 (0.31)
Grand mean of satisfaction over the 16 learning outcomes (SD)	4.22 (0,36)	2.99 (0.51)	4.66 (0.34)	3.46 (0.45)
Mean of age (SD)	54.97 (17.16)	44.21 (16.96)	57.29 (18.24)	47.28 (19.98)
Size of settlement (%)				
Village	2.8	2.8	7.4	3.4
Town	32.2	21.4	31.3	15.9
City	65.0	75.8	61.3	80.7
Level of education* (%)				
Primary school	8.2	4.4	17.8	3.4
Secondary school	67.8	64.3	65.0	64.1
College/university	24.0	31.3	17.2	32.4
Gender (%)				
Male	52.1	36.9	49.1	66.2
Female	47.9	63.1	50.9	33.8
Frequency of doctor visits (%)				
Maximum two times a year	24.6	28.2	23.1	30.3
Several times a year	30.9	36.1	24.1	39.3
Once a month	34.4	29.0	35.3	22.1
Once a week	6.6	3.6	8.8	5.5
Several times a week	3.5	3.2	8.8	2.8

* We handled the patients’ answers who marked ‘other’ category in the variable of level of education as missing data in the cluster analysis

The table shows the descriptive results (means, ratios) of the four-clustered non-hierarchical k-means cluster analysis. The analysis shows that the different clusters have different characteristics and ratings of importance and satisfaction. Cluster 3 has the highest importance and satisfaction rates. Cluster 4 has the lowest importance rates and Cluster 2 the lowest satisfaction rates

Participants of Cluster 1 (N = 317) considered all 16 aspects the third most important on average and had the second highest level of satisfaction. They were on average 55 years old, lived in smaller settlements (village and town) and had lower educational attainment (primary school). The proportion of genders was balanced. Individuals in this cluster had been regularly seen (once a mouth or several times a year) by a doctor in the past year. Individuals in Cluster 2 (N = 252) on average considered all 16 aspects the second most important, but they were the least satisfied with the work of the doctor based on the given criteria. This cluster contained the youngest patients (aged 44 on average). Majority of them lived in bigger settlements (town and city), typically had higher educational attainment. In this cluster the number of females was predominant, and they did not often see a doctor (majority of them several times a year). The level of importance and satisfaction was the highest in Cluster 3 (N = 377). These individuals were the oldest (aged 57 on average), most of them came from small settlements (village or town, their educational attainment was the lowest. The gender proportion in the cluster was equal and they had been to the doctor most often (majority of them once a month and these individuals visit the doctor once a week and several times a week) in the past year. Participants of Cluster 4 (N = 145) had rated the learning outcomes the least important and had the second lowest level of satisfaction. They were 47 years old on average. These individuals came from the cities in the highest proportion, had the highest educational attainment and the proportion of male individuals is predominant here. They met the doctor the least frequently (maximum two times a year) in the past year.

## Discussion

This study examined the importance of learning outcomes in relation to the rate of satisfaction from the patients’ point of views. Patients rated the learning outcomes overall highly important but also gave high satisfaction scores for the implementation of these outcomes in everyday healthcare. The importance and satisfaction rates were near to each other, which imply that the learning outcomes are carried out by doctors nearly in the extent, how important patients think they are. However, the cluster analysis showed that patients’ responses were also influenced by their socio-demographic background. Younger patients in cluster 2 and 4 rarely visited doctors and were the least satisfied with the learning outcomes. Elder patients (in cluster 1 and 3) who visited the doctors more often were the first and second most satisfied respondents. The importance of the learning outcomes was also varied across clusters. The results of the cluster analysis may generally suggest that doctors must pay attention to the patients differently according to their generational needs. It may also be plausible to assume that doctors had not been entirely adapting to the expectations of younger patients since they were the least satisfied. This suggests that doctors should develop their skills along with the changes of the generations as well and implies that practicing as a doctor requires skills which support to be able to treat patients with human dignity, fully inform and handle patients as equals, and give information suitable to the patient (qualification, cultural background, cognitive state). These findings are in line with the report of the General Medical Council, which also emphasizes that patients must be able to trust doctors and to justify this trust doctors must show respect for human life [5]. The Dutch Blueprint also made an emphasis on treating and considering patient as a complete and unique person beyond the professional requirements [4]. Furthermore, the results of the cluster analysis support the widely accepted view that medical education has a major role to play in the training of future doctors by the content and conduct of an educational program and directing students’ attention on the different needs and sociodemographic variables of patients [3].

In the gap matrix, despite high satisfaction scores, the negative values indicated that the satisfaction of patients’ needs were not met in medical care. Although patients hardly questioned the timely theoretical and practical knowledge and the professional practice needed for the everyday work of the doctors, but we recorded bigger gaps (tension) in case of some learning outcomes (e.g. fully informing patients about their diseases, giving information suitable to the patients’ qualification, level of education, cultural background, cognitive state and especially in case of respecting human dignity of the patients and their relatives during patient care). On the one hand, they are legally determined (e.g. the right to information), on the other hand some of them are moral principles which are unquestionable in health care (e.g. human dignity). Based on the results we cannot conclude that these rights, or principles are violated but the bigger gaps emphasize that we need to pay particular attention to these learning outcomes during medical education and health care. Thus, these results underlie the fact that these learning outcomes must get more impact during medical education and clear expectations support improving patient care beyond the immediate professional concerns as suggested by Jennings at al and Coulter’s studies [7, 8]. In line with these findings and based on the responsibility of the medical schools regarding changing the curriculum emphasized in AAMC report [3], at the university where the present study was carried out, steps have been taken to ensure that these results have a concrete impact on the curriculum and the topics of the teacher training courses. Varga et al’s analysis on the importance and delivery rate of the learning outcomes in Hungarian medical education [6] resulted in the conclusion that teachers do not deliver the analyzed learning outcomes as much as their importance rates. Besides the learning outcomes our study also revealed that teachers are not as aware of the importance of learning pedagogical skills as students estimated they should [6]. Calderhead also highlights that systematic collaborative research would be needed to guide the development of a more theoretically and empirically grounded performance-based approach to teacher education [10].

It is supported by studies that having strong pedagogical knowledge and competence can support the delivery of the learning outcomes on a higher level [10–12]. The answers of patients highlight the fact that besides professional knowledge other learning outcomes are also important in health care which should have been emphasized in medical education and medical universities must ensure that before graduation a student had the opportunity to learn them. The lack of pedagogical knowledge shown by the students’ rates on the need for the development of teachers in pedagogical knowledge can also play an important role in the delivery of learning outcomes [6]. This impact on teaching has been revealed by several international research as well [13–18]. Hesketh et al. identified the need for further training and support for medical teachers as well. Besides the importance of learning outcomes, their delivery, and the need for pedagogical development was also reported by the 25th AMEE guide regarding how learning outcomes can be measured and how they are defined as factors which ensure the quality of medical education [15, 19]. Measurement evaluation of the knowledge of the students (e.g. courses in appraisal and assessment) and outcome-based education can be key in delivering and checking the efficiency of learning outcomes [15, 19–21]. Moreover, Hesketh et al. highlights that medical teachers’ needs for developing teaching skills can be quite different so training should match the individual needs, too [15].

### Limitations

As the interviews were conducted face-to-face, there was a probability that there were differences between the interviewers (in spite of the training they participated) which can have an impact on the data. The interviewer may always be influenced by his/her paradigms which can guide the behavior of the interviewee in a special direction [22–24]. The understanding and the comprehensibility of the terminology used in the learning outcomes may have caused difficulties mostly among patients with lower qualification and level of education. It can also cause bias that many respondents may tend to avoid expressing negative opinions or making embarrassing comments about doctors, especially during hospital care. Questions referring to a general view on physicians’ behavior can differ from the reality particularly when respondents may not adequately remember the doctors’ behaviors or perhaps their memory of such events may have evolved with time [23]. Another potential limitation of our study is a time bias. That is, patients evaluated medical care in which physicians had learned along learning outcomes that are partly different to those examined in the present study. In the future analysis we could compare the prioritization of the learning outcomes’ rates and their importance in medical education respectively.

A further limitation of the study may be that we asked the patients about their satisfaction and not experience. Although, in general, the answers to the experience and satisfaction scales may be very strongly correlated, yet they may show small differences. However, we had two reasons for using satisfaction scale. On the one hand, previously Varga et al. [6] used these two scales to compare the opinions of medical students and teachers, so the results of the present study can be compared with those of the previous study. On the other hand, the aim of our study was to investigate how patients are satisfied with the fulfilment of learning outcomes set officially by medical universities. Future studies may be interested in comparing patients’ responses to experience and satisfaction scales.

### Generalizability

Our findings have potentially important implications in Hungarian medical education since it revealed that patients’ satisfaction rate with the learning outcomes could be raised to get more satisfied patients. Satisfaction of the patients have impact on compliance, on the doctor-patient relationship and the prospects of remedy [25]. Since achieving the learning outcomes is the final goal of medical education, this finding plays an important goal in medical education. To the best of our knowledge, there has not been any questionnaire prepared internationally analyzing the attitudes of patients towards the learning outcomes. Yazdani and Noghabaei reported on the attitudes of the graduate medical students toward the learning outcomes, but teachers and patients were not involved in this study [26]. This study also suggested the importance of learning outcomes from a quality assurance aspect as well.

Firstly, the study revealed that learning outcomes determined by the Hungarian law are important in medical educations as patients rated them high on a five-point scale. We can conclude that almost all the learning outcomes are rated above a mean of 4.0. Secondly, patients’ satisfaction rates could be improved by giving more emphasis on analyzing their regular feedback on health care and paying more attention on delivery during medical education. Thirdly, we can detect a connection between the learning outcomes taught in medical education and our previous findings that teachers do not feel the need for further development regarding their pedagogical skills as students do. Our suggestion is that focusing more on the teachers’ skills would have resulted in more satisfied patients and would result in more satisfied patients in the future as well. Thus, the better realization of learning outcomes in health care can give an emphasis to the role of medical education and medical teachers and their professional and pedagogical development. Fourthly, building a system of special pedagogical education in medical education based on the feedbacks of the patients would increase their satisfaction. Finally, asking patients about their needs and ideas about the health care system can have a huge implication in changing medical education in the future. Moreover, the motivation for changing the health care system and the teaching methods is being accelerated by the emergence of COVID-19, as patients can have different expectation since the pandemic started. Although the study points out and international research outcomes also support the importance of learning outcomes, there is no published evidence suggesting the same expectation of the students participating in medical education. Due to the lack of this evidence and because the analysis was based only on the teachers’ and patient’s opinions more evidence on the subject is needed.

## Conclusions

The study underlines the results of previous studies [11–15], the importance of learning outcomes and the need of a paradigm shift in medical education from teaching only professional knowledge to future doctors to giving an emphasis to other learning outcomes as well. Besides the need of soft skills in medical education the need for pedagogical skills as part of the training of medical teachers can play an important role in the development of medical education based on the results coming from the patients, who are less satisfied with the learning outcomes than they consider them important.

## Data Availability

The datasets used and/or analyzed during the current study are available from the corresponding author on reasonable request.
